# Exploring the transition to DRGs in Developing Countries: A case study in Shanghai, China

**Published:** 2014

**Authors:** Zhaoxin Wang, Rui Liu, Ping Li, Chenghua Jiang

**Affiliations:** 1 Zhaoxin Wang, Shanghai Tenth People's Hospital, Tongji University School of Medicine, China.; 2 Rui Liu, Shanghai Tenth People's Hospital, Tongji University School of Medicine, China.; 3 Ping Li, Zhou Pu Hospital, Shanghai, China.; 4 Chenghua Jiang, Tongji University School of Medicine, China.

**Keywords:** Diagnosis Related Groups, Payment, Hospitals, China, Hypertension, Coronary Heart Disease

## Abstract

**Objective:** With the success of DRGs (Diagnosis Related Groups) in developing countries, this prospective payment system has been imported into China from the early 21^st^ century. However, DRGs has been struggling and has made little progress since (its adoption in) 2004. This study contributes to the debate on how to bridge the pay-for-service (system/scheme) and DRGs (Diagnosis Related Groups) during the transitional period of payment reform in China.

**Methods: **From 2008 to 2012, sixty regional general hospitals in Shanghai were divided into three groups according to their economic level, and one hospital was picked from each group randomly. After ranking of morbidity, 22130 patients with hypertension or coronary heart disease were chosen as sample. Using multiple linear regression analysis, the inter relationships between the total medical expenses of the inpatients, and age, gender of the inpatients, length of stay, region and economic level of the hospitals were examined.

**Results: **The main findings were (1) Age, LOS and the economic level of treatment location had a statistically significant impact on patients with hypertension or coronary heart disease. However, gender is only a significant factor to patients with coronary heart disease. The results suggested that age, LOS and the economic level of treatment location should be considered in formulating pricing standards for the hypertension patient group. Besides the above mentioned factors, gender should also be considered in formulating pricing standards for the coronary heart disease patient group. (2) Under the premise of limited resources, developing countries should first narrow down to screen for common and frequently occurring diseases, then study the key factors which affect the treatment cost of the diseases.

**Conclusion:** Simplification of the DRGs standard- setting process based on standardized clinical pathways and accurate costing will greatly increase the efficiency of implementing DRGs in the developing world.

## INTRODUCTION

Diagnosis Related Groups (DRGs) are one of the most striking prospective payment systems around the world in recent years ^1^^, ^^2^ As a classification system that groups patients according to (a) principal diagnosis, (b) type of treatment, (c) age, (d) surgery, and (e) discharge status ^3^^, ^^4^, prospective payment system have gradually become the principal means of reimbursing hospitals in most developed countries, such as US, Germany and Australia ^5^. Under the prospective payment system, hospitals are paid a set fee for treating patients in a single DRG category, regardless of the actual cost of care accrued for the individual, as to create incentives for hospitals to control cost, to reduce the LOS of patients and to increase number of inpatient admissions.^6^^, ^^7^

Successful experiences of DRGs in developed countries have encouraged developing countries to adopt the system. Since 2000, countries such as Brazil, Mexico and Iran have introduced DRGs reform in order to control cost and to increase hospital efficiency ^8^^, ^^9^. However, those countries met varying degrees of difficulties during the implementation of the policy. For example, Iran developed an interest in casemix funding of hospital as part of a major health financing reform in 2003^10^. But the usefulness of DRG information for either management or funding arrangement is still under question due to the poor quality of hospital data.^11^

In fact, DRGs is not a new term to China^12^. As early as the beginning of the century, some of China’s relatively economically developed provinces have started the exploration of DRGs ^13^^, ^^14^. For example, Shanghai has experimented with a prospective payment system whereby a reimbursement cap is imposed on each Diagnosis-Related Group (DRG) in 2004. This experimentation includes fifteen types of different diseases ^15^. However, the DRG mentioned above is greatly different from the foreign DRGs ^16^: a) it adopts survey results on hospitalization costs for corresponding diseases, b) it bases on the average medical insurance cost of the past few years, c) it formulates insurance payment standard for a single disease. In a word, the payment standard merely sets a maximum payment limit for a particular disease. The reason why China’s reform attempt did not fully introduce foreign experience was because during 2004, objective conditions for the implementation of DRGs were not mature yet in the following three aspects even in China’s most developed city, Shanghai: 1) hospitals had low information technology. Daily practice mostly relied on manual records with poor normality and comparability; 2) medical recording was not standardized, leading to limitation in the collection of patient related information and incomplete recording of paper materials; 3) the payment for services was deeply ingrained and many doctors feared that it may impact their revenue, thus there was resistance to reform.

China’s new medical reform was launched in 2009, which had four important parts^17^ : public health, medical services, basic drugs and medical insurance. Payment innovations have become the most prospective direction for medical insurance reform in these years ^18^. Currently in Shanghai, the level of health information technology has been gradually improved. Also, medical record keeping and diagnosis coding have been more standardized, which provided a foundation for the formal practice/ implementation of DRGs ^19^. However, due to China’s complex social and economic background, and uneven development across regions, the time was not yet ripe for the formulation of a national payment standard ^20^. Since DRGs cannot be achieved overnight in developing countries, a step by step realization approach is required.

In the context of limited resources, the reasonable grouping of cases is difficult to cover all diseases. Hence first of all, it is necessary to analyze the common and frequently occurring diseases of a region within a period of time. Secondly, target the first few high incidence diseases ranking on top of the disease spectrum, then analyze the factors associated with the medical expenses of these diseases (such as demographic characteristics of patients, disease diagnosis, treatment, and other relevant factors). This can provide a good prediction on the feasibility of formulating payment standards for each disease group. This approach can also provide developing countries with relatively low level of healthcare technology a theoretical framework to implement DRGs during the transition from the past payment system.

## METHODS

***Sites and Sampling: ***This research was a retrospective examination of data from 32,000 hospital stays at 60 regional general hospitals from 2008 to 2012 in Shanghai. We divided the 60 hospitals into three groups by economic level, and then picked one hospital from each group randomly. All discharges from the selected hospitals were included in the database. We chose to start with the data of 2008 because it was the earliest year that cost data was available and sufficient, and 2012 because it contained the most recent data when we performed our analysis. Shanghai has been promoting information technology gradually since 2007. While the development on medical recording and other important foundation for the implementation of DRGs in the district general hospitals is uneven, in general the circulatory diseases clinical department has the fastest growth on the degree of standardization in clinical pathways and medical recording. Therefore, this study selected 40,019 circulatory system discharged cases as sample for analysis from 2008 to 2012. Among the types of diseases, coronary heart disease and hypertension have the highest incidence rates, the number of cases being 16,235 (40.4%) and 5,895 (14.7%) respectively. As the proportion of the remaining cases occurred less than 10%. 22,130 discharged patient cases from 2008 to 2010 are finalized as the total sample for this study. 

Because the sample did not contain identifiable private information, the Institutional Review Board at Tongji University did not consider this analysis to be human subjects research. 

***Participants: ***Patients in our study were admitted to hospitals from 2008 to 2012 and were discharged with a principal diagnosis of hypertension or coronary heart disease. All these diagnoses were the top three diseases on the spectrum of disease in five years. 

***Data and*** ***Statistical*** ***Analysis: ***For each patient, we included the factors of age, gender, length of stay, principal and secondary diagnoses, and the total medical expenses. Hospitals were categorized by region and economic level. In our primary analysis, we used multiple regression model to find correlations between the total medical expenses and traits of inpatients and hospitals during 2008 to 2012 for each of the five principal diagnosis at sampled hospitals. We held covariates constant at their overall sample means. The model included patient factors—age, gender, length of stay, principal and secondary diagnosis, the total medical expenses—as well as hospital factors—region and economic level. All analysis were carried out using SPSS version 17.0. All *p* values less than 0.05 were considered significant. 

## RESULTS

***Patient and Hospital Characteristics: ***The data for age and gender of the 22,130 hypertension or coronary heart disease discharge cases were derived from case records. Among the discharged cases, there were less than 50% of male cases. By dividing the cases into five groups using 10 years as an interval, there was a yearly decrease in proportion of cases for patients age 50 below, and a trend of yearly increased for patients age 81 above, which was associated with improvements in the level of chronic disease prevention and health standards of Shanghai residents. To analyze whether the expenses for medical treatment is related to the economic environment of the healthcare provider hospital, the study also divided the hospitals into three groups of high, medium and low according to each hospital’s level of economic environment. (Table-I).

**Table-I:** Descriptive statistics of patients’ regional general hospitals from 2008 to 2012 in Shanghai

*Characteristics*	*2008*			*2009*			*2010*			*2011*			*2012*	
	*N*	*Percent(%)*		*N*	*Percent(%)*		*N*	*Percent(%)*		*N*	*Percent(%)*		*N*	*Percent(%)*
Total	4090	100		4961	100		4729	100		3770	100		4580	100
Age (year)														
under 50	240	6		181	4		201	4		130	3		90	2
51-60	533	13		371	7		432	9		280	7		32	7
61-70	487	12		799	16		566	12		480	13		638	14
71-80	1210	30		1710	35		1680	36		1324	35		1440	31
above 81	1621	40		1900	38		1850	39		1556	41		2110	46
Male	1609	39		2001	40		1880	40		1461	39		2120	46
Economic level of environment^a^														
low	1089	27		1691	34		1929	41		850	23		410	9
mid	1731	42		2402	48		1644	35		2110	56		1720	38
high	1270	31		868	18		1156	25		810	21		2450	53

aLow, high or mid: Economic level of areas.

**Table-II:** The total medical expenses of inpatients with hypertension or coronary heart disease from 2008 to 2012

		*2008*		*2009*		*2010*		*2011*		*2012*	
		*H* a	*CHD* b	*H*	*CHD*	*H*	*CHD*	*H*	*CHD*	*H*	*CHD*
**Total charge**	N	1309	2781	1459	3502	1039	3690	815	2955	1272	3308
	Minimum	1313.58	205.70	290.40	213.93	808.02	581.87	1799.49	1304.05	1906.68	235.47
	Maximum	18566.57	26167.74	54648.05	31400.31	18494.49	39952.41	21850.68	24222.95	20193.28	32455.36
	Mean	5638.55	6075.16	6629.45	6929.52	6428.06	7544.60	7338.75	8445.70	8365.75	10011.70
	Std. Deviation	3035.07	3770.90	5383.92	4571.11	3622.30	4772.54	3866.42	4254.63	3637.19	4645.44

Monetary unit of all charges is in Renminbi (RMB),

a H: hypertension,

b CHD: coronary heart disease.

**Table-III:** Independent Samples Test of hypertension and coronary heart disease

		*Levene's Test for Equality of Variances*		*t-test for Equality of Means*				
		*F*	*Sig.*	*t*	*df*	*Sig. (2-tailed)*	*Mean Difference*	*Std. Error Difference*
Total charge	Equal variances assumed	14.680	.000	4.519	2211	.000	980.93276	217.05445
	Equal variances not assumed			4.758	1158.099	.000	980.93276	206.14649

**Table-IV:** The results of regression model of hypertension and impact factors

*Model*	*Unstandardized Coefficients*		*Standardized Coefficients*	*T*	*Sig.*
	*B*	*Std. Error*	*Beta*		
(Constant)	-2025.437	611.807		-3.311	.001
age	43.004	8.398	.136	5.121	.000
LOS^a^	470.399	16.590	.740	28.354	.000
gender	104.047	224.574	.012	.463	.643
high^b^	1563.142	286.359	.162	5.459	.000
mid	476.814	248.404	.057	1.920	.055

a LOS: length of stay,

b High or mid: Economic level of areas, R Square=0.615, Adjusted R Square=0.612

**Table-V:** The results of regression model of coronary heart disease and impact factors

*Model*	*Unstandardized Coefficients*		*Standardized Coefficients*	*t*	*Sig.*
	*B*	*Std. Error*	*Beta*		
(Constant)	-2681.777	591.573		-4.533	.000
age	47.183	7.481	.108	6.307	.000
LOS	588.240	14.004	.712	42.004	.000
gender	-339.481	160.611	-.036	-2.114	.035
high	1350.681	208.863	.135	6.467	.000
mid	191.161	195.310	.020	.979	.328

a LOS: length of stay,

b High or mid: Economic level of areas, R Square=0.546, Adjusted R Square=0.544

Hypertension and coronary heart diseases have been the most frequently occurring diseases in the district hospital departments of Shanghai since 2008. In order to analyze whether the primary diagnosis impacts the differences in medical expenses, the study first depicts the change in expense for hypertension (H) and coronary heart disease (CHD) in five years. Although the maximum and minimum medical expenses for the two diseases fluctuated yearly, there was an upward trend. The mean expense for hypertension had increased from RMB 5,638.55 to RMB 8,365.75 in five years. To further analyze whether the medical expenses of the two diseases are different, the study applies t-test for Equality of Means and found p<0.05. The difference was statistically significant, showing that the primary diagnosis had a significant impact on the medical cost of discharged patients. (Table-II & III)

For Table-IV and V, with the inpatients medical expenses as the dependent variable, the inpatients’ age, gender and length of hospital stay as independent variables, and the economic level of treatment location as dummy variable (regions with higher economic level set at high, with moderate economic level set as mid, with lower economic level set as constant), results from multiple linear regression were as follows: targeting on the regression model of patients with coronary heart disease, overall the model had a high goodness of fit, with R Square= 0.615. Despite that the Unstandardized Coefficients of gender and regions with moderate economic level were not statistically significant, the impact of coronary heart disease patients’ genders on medical expenses was statistically significant, with P value less than 0.05. The Unstandardized Coefficients of regions with moderate economic level alone was not statistically significant. For the medical expenses of patients with hypertension, age, gender, length of stay and economic level had a greater impact (with P value less than 0.05).

## DISCUSSION

DRGs has become the global trend in medical payment reform. This prospective payment system is not the privilege of developed countries, many developing countries (like Iran) have begun to adopt DRGs. However, due to the level of medical standards and economic constraints, the direct translation of foreign experience is undesirable. It is particularly important for developing countries to handle the transition to DRGs from its current medical payment system. Analyze the factors that affect the medical expenses of specific diseases. Simplify the DRGs standard- setting process.

According to CISS, the DRGs standard setting process includes 1) definition of the objectives of the DRG system, 2) analysis of feasibility, 3) choosing the classification system for patients, 4) level of implementation, 5) integration of the components, 6) operation of the system, 7) consolidation in the long term. Although the implementation of DRGs is very standardized in developed countries, it is difficult for developing countries with limited resources to directly adopt the seven steps foreign experience when implementing DRGs. This is the reason why Shanghai’s adoption of DRGs is not progressing in the past ten years since 2004. To implement DRGs successfully, developing countries such as China have to prioritize its steps. Starting with definition of the objectives of the DRG system, the most common diseases in each disease group should be first targeted so as to formulate pricing standards for each disease group and increase the feasibility of implementing DRGs.

For example, the research has first confirmed that hypertension and coronary heart diseases are the most common circulatory diseases in Shanghai. Then through the sample analysis, gender is found to be an unimportant factor for hypertension medical expense, but affects the medical expenses of coronary heart disease patients. Therefore, gender should be included in formulating payment standard for hypertension, but not for coronary heart disease. Using the above as an example, targeted disease group pricing is possible through considering the inclusion or exclusion of related factors.

***DRGs will*** ***become*** ***an important direction for China’s medical reform: ***In hypertension and coronary heart disease, which are the two highest incidence circulatory diseases, age and length of stay are common influencing factors for medical expenses, thus these two factors should be considered in disease grouping. In particular, length of stay was positively correlated with the expense of medical treatment, which brings hope to resolving the two current medical dilemmas in developing countries: high medical expenses and bed turnover inefficiency. These two dilemmas have caused the headache of expensive and difficult medical treatment in China. The good news is a large number of studies have confirmed that DRGs can effectively improve bed turnover rate and reduce hospital bed days. Hence DRGs would be a major direction for China’s medical reform.

***Limitations of the study and directions for future development: ***There are two limitations in this study. On one hand, the sample size is limited thus cannot represent the distribution of disease cost in the whole population. If a larger sample is available, the analysis of impact factors for specific diseases would be more accurate. Also, data mining techniques can be introduced for the cost of disease clustering based on key factors, thereby to develop more accurate pricing standards for each disease group. On the other hand, Shanghai, as one of China’s most developed cities, has the highest economic and medical technological level in the nation, thus the results of this study cannot be generalized. This study mainly provides fellow colleagues research an idea on the way to introducing prospective payment system during transition from past payment system. For future studies, the direct application of research results has to be improved. 

## CONCLUSION

Conditions in developing countries are not up to the management requirements to fully realize DRGs. However, in the context of limited resources, starting with screening for common and frequently occurring diseases, studying the limited diseases within each disease group and the key factors influencing medical expenses of these diseases, as well the simplification of DRGs standard- setting process based on standardized clinical pathways and accurate costing, will greatly increase the efficiency of implementing DRGs in developing countries. 

## Notes

***Source of funding:*** This work was supported by the Fundamental Research Funds for the Central Universities (2012KJ017), Research Funding from the Health bureau of Shanghai((20124y182& XYQ2013111), Shanghai Postdoctoral Scientific Program(13R21416600) and China Postdoctoral Science foundation (2013M531223).
